# The “grey” digital divide in older adults during COVID-19 in Germany: Who is most at risk?

**DOI:** 10.1093/eurpub/ckac130.059

**Published:** 2022-10-25

**Authors:** N Pedrós Barnils, B Schüz

**Affiliations:** 1 Prevention and Health Promotion, Bremen University, Bremen, Germany; 2 Digital Public Health, Leibniz Science Campus, Bremen, Germany

## Abstract

**Background:**

During the COVID-19 pandemic, physical distancing was a recommended public health measure. For some older adults, distancing has not only been physical but also social due to a lack of access to the internet - either due to consciously opting out or lack of resources. This study aims to assess who is most at risk of not having access to the internet and the associated negative mental and physical health outcomes.

**Methods:**

Participants were drawn from the 2020 German Ageing Survey (DEAS) in June and July 2020, and include community-dwelling adults above 45 years old (N = 4,823; 56.5% response rate). Two complementary analytic approaches were used to identify lack of access: logistic regression (LR) and Classification and Regression Tree (CART) analysis, using social indicators as predictors. LR provides information about main effects of the predictors; CART, through an exploratory, non-parametric procedure, illustrates the mathematical relationship of the variables.

**Results:**

CART analysis revealed that the strongest discriminating factor for internet access was being over or under 75 years old (n = 3,131 Pr = 0.075 vs n = 1,545 Pr = 0.385). Moreover, for older individuals high education was a protective factor (n = 739 Pr = 0.260 vs n = 805 Pr = 0.499), while for younger individuals a monthly income of 2,000€ set the internet access cut-off point (n = 2520 Pr = 0.0504 vs n = 611 Pr = 0.177). Logistic regression revealed that gender (OR = 1.50; pv < 0.001), education (OR = 0.36; pv < 0.001), monthly income (0.93; pv < 0.001) and region in Germany (West-East) (OR = 2.42; pv < 0.001) explain 29.40% of internet access’ variance. Results are preliminary.

**Conclusions:**

In times of forced physical distancing, the “grey” digital divide increases the vulnerability of disadvantaged groups. CART analysis helped identify groups at higher risk of not having access to the internet and yields the ground for tailored public health interventions.

**Key messages:**

- The strongest discriminating factor for internet access in Germany during the COVID-19 pandemic was being over or under 75 years old. For younger individuals a monthly income of 2k € set the threshold.
- The “grey” digital divide affects individuals over 75 years old with middle or low education the most, increasing the risk of associated negative mental and physical health outcomes.

